# Utility of cardiac magnetic resonance in assessing right-sided heart failure in sarcoidosis

**DOI:** 10.1186/1471-2342-13-2

**Published:** 2013-01-11

**Authors:** Jacob Lønborg, Michael Ward, Anthony Gill, Stuart M Grieve, Gemma A Figtree

**Affiliations:** 1 North Shore Heart Research Group, Kolling Institute of Medical Research, University of Sydney, Sydney, Australia; 2 Department of Cardiology, Rigshospitalet, Copenhagen, Denmark; 3 Department of Cardiology, Royal North Shore Hospital, St Leonards, NSW 2065, Australia; 4 Department of Radiology, Royal Prince Alfred Hospital, Sydney, Australia; 5 Department of Pathology, Royal North Shore Hospital, Sydney, Australia

**Keywords:** Myocardial sarcoidosis, Cardiac magnetic resonance, Heart failure, Cardiomyopathy

## Abstract

**Background:**

Cardiac involvement in sarcoidosis is associated with a poor prognosis. In patients with right sided heart failure, differentiating between cor-pulmonale, or cardiac sarcoidosis has important implications to management.

**Case presentation:**

We present the case of a patient with severe but stable pulmonary sarcoidosis and new onset right sided heart failure despite only mild elevations of pulmonary artery pressure. CMR demonstration of extensive right ventricular fibrosis with associated dilatation and hypokinesis was a key finding for prognosis and management of the patient.

**Conclusion:**

Cardiac magnetic resonance (CMR) is the preferred investigation in the diagnosis of cardiac sarcoidosis, allowing assessment of myocardial inflammation and fibrosis, as well as function, in a manner not matched by other technologies.

## Background

Sarcoidosis is a multi-organ disease characterised by the formation of granulomas. The lungs are most commonly affected. Although cardiac involvement is clinically detected in only 2% of patients, underdiagnosis is a major issue of considerable clinical importance. Recent autopsy data suggest that the heart is, in fact, involved in 20-25% of patients [1], and is directly responsible for up to 25-80% of deaths attributed to sarcoidosis [2].

In patients with identified cardiac sarcoidosis the left ventricle (LV) is involved in 98% and the right ventricle (RV) in up to 40%, usually in the context of biventricular involvement [3]. Cardiac sarcoidosis affecting predominantly the RV is rare. In a patient with severe, but stable pulmonary sarcoidosis, we illustrate the important role of CMR in the diagnosis and management of new onset RV failure. CMR is able to identify granulomatous infiltration of the RV, providing evidence for sarcoidosis as the cause of primary RV failure rather than cor pulmonale as the cause of the patients presentation.

## Case presentation

**Figure 1:**
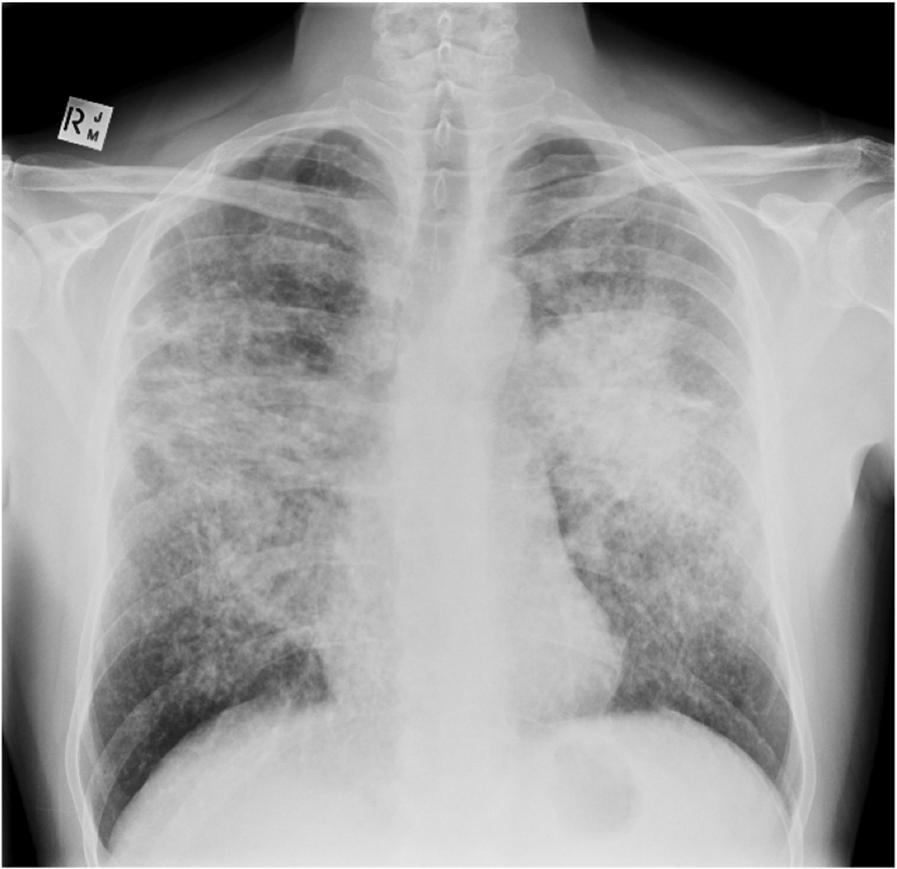
**A frontal chest x-ray showing changes consistent sarcoidosis. **There is bilateral bulky hilar and paratracheal lymphadenopathy together with extensive, confluent reticulonodular opacities with a characteristic distribution in the mid and upper zones.

**Figure 2:**
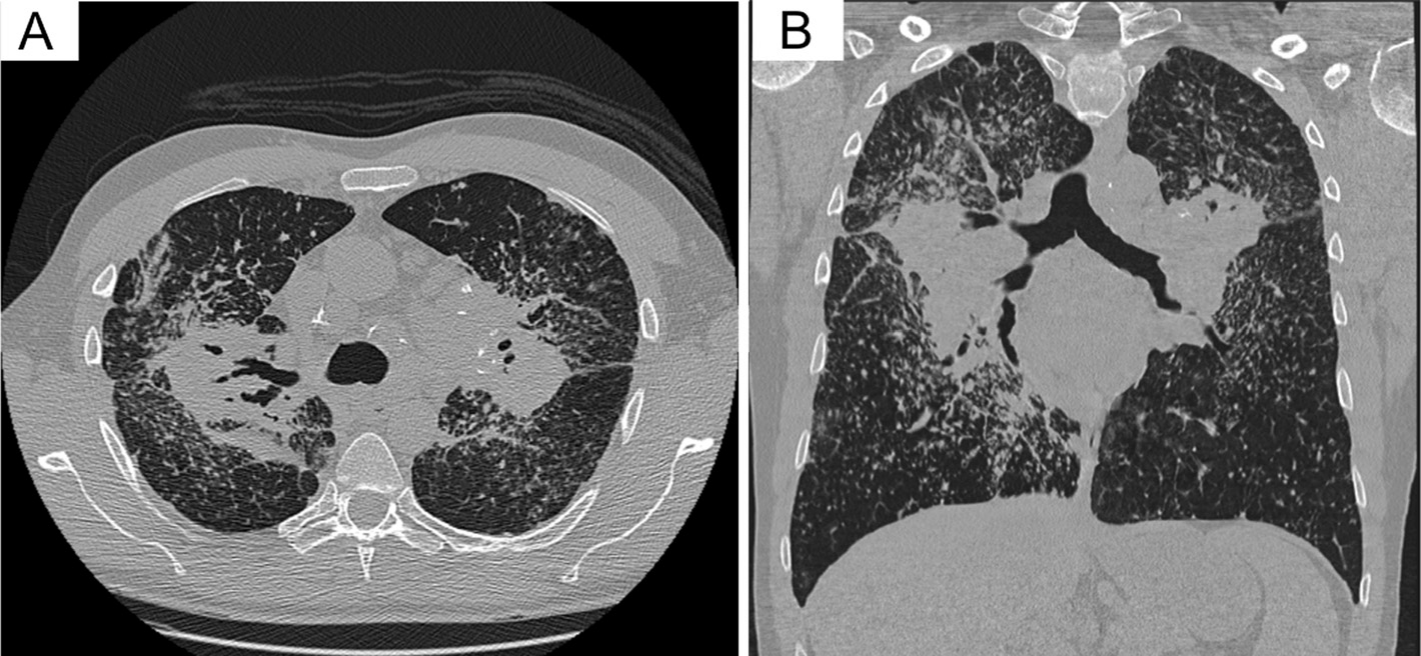
**Selected axial (A) and high resolution coronal (B) reformats from a non-contrast helical CT shows striking bilateral bulky hilar and mediastinal adenopathy. **Mass-like fibrosis is encasing the hila and central bronchi causing traction bronchiectasis. There are several partially calcified hilar lymph nodes. There are also extensive perivascular and perilymphatic nodules throughout both lobes with relative sparing of the lower zones and right upper lobe bullae.

**Figure 3:**
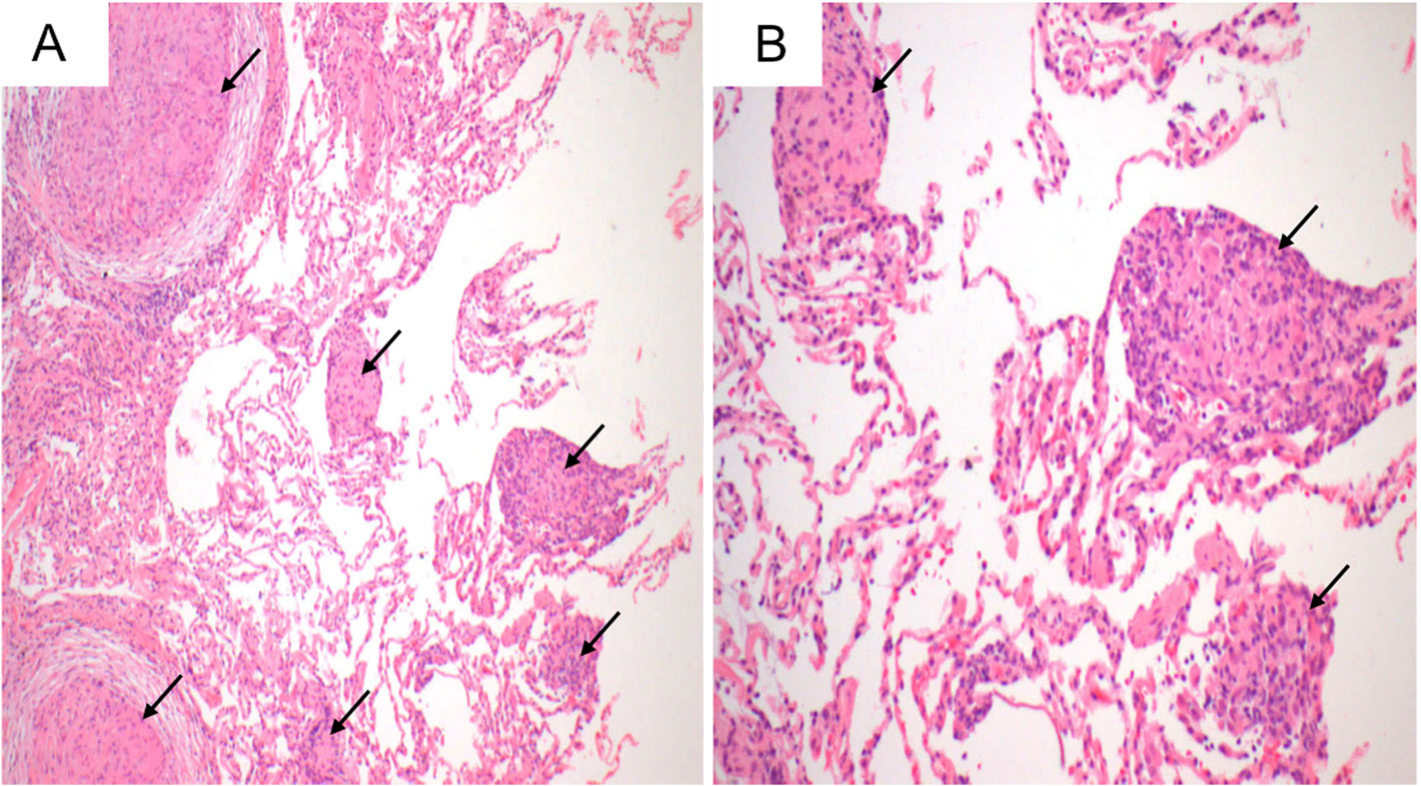
**Histology H&E images, original magnifications 200x (A) and 400x (B), of specimen from the transbronchial lung biopsy. **It identified numerous well-formed, non-necrotising epithelioid granulomas (arrows) at both low (**A**) and intermediate (**B**) power.

A 42-yr old man initially presented with exertional dyspnoea and cough. He had no previous medical history of significance and was a non-smoker. The clinical examination and biochemistry were unremarkable, except for elevated serum angiotensin converting enzyme (ACE). Chest x-ray showed bulky hilar lymphadenopathy with extensive reticulonodular and mass like opacities in both lungs with a middle zone predominance (Figure 1). A non-contrast computed tomography (CT) scan of the thorax demonstrated numerous nodules in a perilymphatic distribution (subpleural and peribronchovascular) together with perihilar conglomerate masses (Figure 2). Several enlarged partly calcified hilar, paratracheal and subcarinal mediastinal lymph nodes were evident. Histopathology of a transbronchial lung biopsy showed numerous well-formed, non-necrotising epithelioid granulomas (Figure 3), but tests for bacilli, fungi and tuberculosis were negative. Based on clinical, radiological and pathological findings the diagnosis of pulmonary sarcoidosis was made.

**Figure 4:**
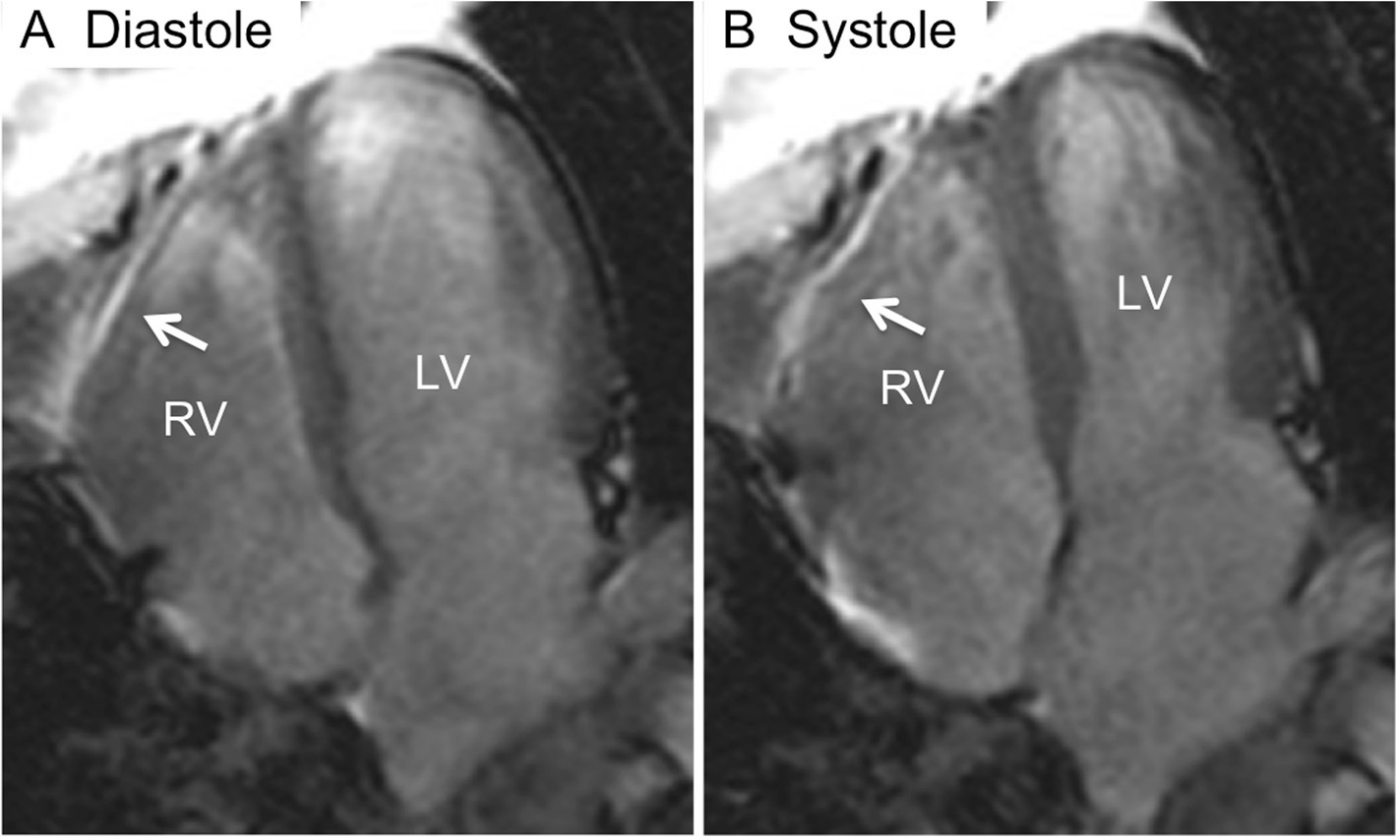
**Functional (cine) cardiac magnetic resonance images in the four-chamber image plane in diastole (A) and systole (B). **The images show poor contraction of the right ventricle (RV) free wall (arrows in **A** and **B**), but normal contraction of the left ventricle (LV).

**Figure 5:**
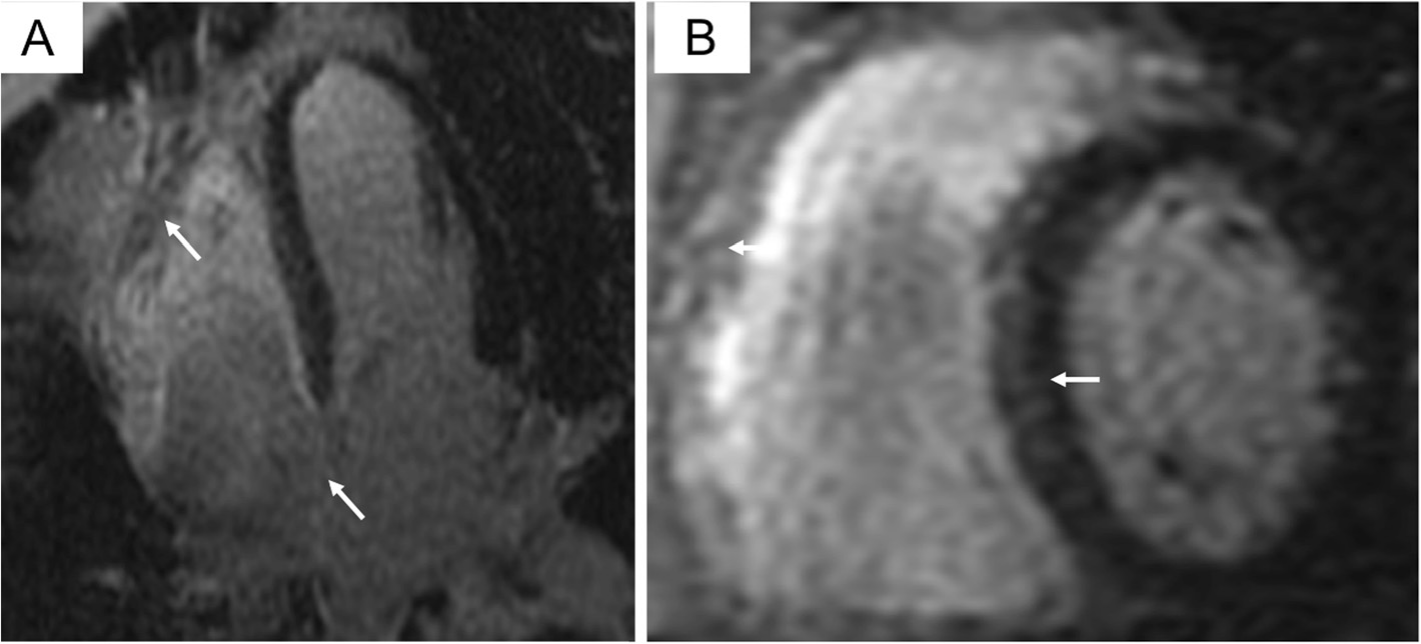
**Delayed enhancement cardiac magnetic resonance images in the four-chamber (A) and short axis (B) image plane. **The images show a patchy pattern of delayed enhancement (arrows) in the subepicardial and mid-wall of the right ventricle free wall, interventricular and interatrial septum. The myocardium of the left ventricle free wall is normal.

Five years after initial presentation the patient represented with dyspnoea and peripheral edema. ACE was within normal limits and electrocardiogram (EKG) was normal. Findings on chest x-ray and CT scan showed minimal change compared to previous imaging. Transthoracic echocardiography showed normal LV function, and a severely dilated RV with moderately impaired contractile function. Pulmonary pressures were only mildly elevated as measured by both echocardiography and right-heart catheterisation. A cardiac magnetic resonance (CMR) scan confirmed a dilated RV with impaired systolic function (Figure 4), RV ejection fraction (EF) was 34% (normal 44–63%). Extensive, patchy delayed gadolinium enhancement was observed in the RV free wall, as well as subtle mid-wall delayed enhancement in the interventricular and the interatrial septum (Figure 5). The CMR findings led to a diagnosis of right heart failure secondary to sarcoidosis infiltration of the RV wall*.* The evidence of RV infiltration by sarcoidosis led to immediate immunosuppressive therapy in accordance with general recommendations for cardiac sarcoidosis, and insertion of an implantable cardioverter-defibrillator (AICD). Follow-up interrogation of the AICD revealed an episode of non-sustained fast ventricular tachycardia.

## Discussion

The diagnosis of cardiac sarcoidosis is often problematic due to non-specific manifestations, as seen in the present case. These findings include non-specific EKG changes, clinical heart failure, abnormal non-specific echocardiography findings, elevated serum ACE and sudden death [1]. A multimodality approach has traditionally been used to determine cardiac involvement [4]. Dyspnoea from LV heart failure may be attributed to primary pulmonary sarcoidosis. Signs of right heart failure may be put down to pulmonary hypertension secondary to pulmonary sarcoidosis and cor pulmonale. The diagnosis of cardiac sarcoidosis predominantly involving the RV may be even more problematic and may need to be distinguished from arrhythmogenic RV cardiomyopathy. Abnormalities on diagnostic tests such as EKG and echocardiography do not differentiate cardiac sarcoidosis from other cardiac diseases. However, in a patient with sarcoidosis any abnormal findings on EKG or echocardiography should lead to further investigations.

Recently, advanced imaging techniques have been used to identify and characterise cardiac sarcoidosis, with both positron emission tomography (PET) and CMR shown to be superior to the previously recommended scintigraphy [5,6]. CMR has a sensitivity of 100% and a specificity of 78% for the diagnosis of cardiac sarcoidosis [6]. CMR allows for accurate assessment of cardiac function and comprehensive tissue characterisation. Functional CMR images assess ventricular volumes, function and focal wall motion abnormalities; T2 weighted black blood images can be used to identify oedema and the presence of active inflammation; and delayed gadolinium enhancement images detect scarring with great accuracy [7]. New emerging pre- and postcontrast T1 mapping CMR techniques may provide information regarding interstitial fibrosis and extracellular matrix volume [8]. However, these new techniques are not yet performed routinely in clinical practice. The pattern of oedema or myocardial late enhancement is used to differentiate between non-ischemic and ischemic scarring. Typical cardiac sarcoidosis lesions seen with CMR are focal mid-myocardial or sub-epicardial scarring, whereas sub-endocardial lesions, typical for coronary artery related infarction, is rare with sarcoidosis [6]. CMR is the optimal method of imaging the RV, both in regards to its contractile function, as well as the presence of sarcoid infiltration with late enhancement, as demonstrated by this case. Serial CMR is also useful in the evaluation of anti-inflammatory treatment [6]. The high sensitivity of 18F-FDG PET for active inflammatory lesions is partially countered by the reduced specificity of 18F-FDG in the heart due to the high metabolic rate of the myocardium. However, it is very likely that a complementary role for PET and CMR in the Clinic will be defined in the near future.

Limitations of CMR include significant claustrophobia in up to 5% of patients and contraindication to gadolinium administration in patients with impaired renal function, and inability to image patients with non-MRI compatible pacemakers or AICDs. Furthermore, although CMR is superior to echocardiography in imaging RV structure and function, the thin walled nature of the RV makes delayed enhancement difficult to discriminate from artefact. This is even more problematic when using T2 based imaging sequences to identify oedema in the RV.

Although patients with cardiac sarcoidosis are at risk of adverse cardiac events including ventricular tachycardia (VT) and CMR is predictive of this [9], the role of prophylactic AICD is not well defined. It is clear that all patients with established VT should receive an AICD and anti-arrhythmic therapy. The patient presented received an AICD due to the extensive nature of RV involvement, with associated dilatation and impaired systolic function. The recorded episode of rapid ventricular tachycardia suggests that this was an appropriate management decision.

## Conclusion

CMR is critical in differentiating the cause of new onset right heart failure in a patient with stable pulmonary sarcoidosis, but no significant pulmonary hypertension. Major cardiac events, including sudden cardiac death often occur in patients despite normal EKG and echocardiography. The excellent sensitivity of CMR, its ability to predict cardiac events, and its lack of ionizing radiation has led to the proposal by many experts that CMR should be part of the routine work-up in any patient with sarcoidosis.

## Consent

Written informed consent was obtained from the patient for publication of this case and any accompanying images. A copy of the written consent is avaliable for review by the Series Editor of this journal.

## Abbreviations

ACE: Angiotensin converting enzyme; CMR: Cardiac magnetic resonance; CT: Computed tomography; EF: Ejection fraction; EKG: Elektrocardiogram; LV: Left ventricle; PET: Positron emission tomography; RV: Right ventricle.

## Competing interests

There were no competing interests.

## Authors’ contributions

JL participated in the concept and design; contributed to the imaging analysis; and drafted the manuscript. MSG, MW and GAF participated in the design and concept; contributed to the imaging analysis; and revised the manuscript critically. All authors have read and approved the final manuscript.

## Pre-publication history

The pre-publication history for this paper can be accessed here:

http://www.biomedcentral.com/1471-2342/13/2/prepub
